# Supplementary material for: Flexible theta sequence compression mediated via phase precessing interneurons
Source: eLife. 2016 Dec 8;5:e20349. doi: 10.7554/eLife.20349 (PMC5245972; doi:10.7554/eLife.20349)
Supplement: Figure 1—source data 1. — Our model is the first to successfully explain speed-modulation of precession frequency, two-dimensional phase precession and 360 degrees of phase precession without introducing unobserved circuit components, directionally modulated external inputs or inputs with speed-modulated oscillation frequencies. DOI: http://dx.doi.org/10.7554/eLife.20349.004 [file elife-20349-fig1-data1.docx]

Figure 1-source data 1

Table comparing the proposed model to previous models of phase precession. Our model is the first to successfully explain speed-modulation of precession frequency, two-dimensional phase precession and 360 degrees of phase precession without introducing unobserved circuit components, directionally modulated external inputs or inputs with speed-modulated oscillation frequencies.

| **Model** | **Core cell type** | **Core mechanism** | **Inputs** | **Output phase range** | **Speed- modulation of frequency** | **Precession in 2D** | **Conflicting observations / limitations** |
| --- | --- | --- | --- | --- | --- | --- | --- |
| *O'Keefe and Recce, 1993; Burgess et al. 2007* | Pyramidal cell | Interference between oscillations with differing frequencies. | Reference oscillation + speed-  dependent oscillator. | 360 | Speed-  modulation of frequency of oscillatory input. | Requires multiple speed- and direction- dependent oscillators. | Place cells typically have a single firing field. Rate and phase codes can be independent (Huxter et al., 2003, Schlesinger et al., 2015). |
| *Mehta et al. 2002* | Pyramidal cell | Excitation-  dependence of spike latency, asymmetric input. | Asymmetric excitatory input + reference oscillation. | 180 | Timescale of asymmetric excitatory input. | Requires directionally modulated input asymmetries. | Phase precession > 180 degrees. Rate and phase codes can be independent (Huxter et al., 2003, Schlesinger et al., 2015). |
| *Harris et al. 2002* | Pyramidal cell | Excitation-  dependence of spike latency, spike frequency adaptation. | Excitatory drive + reference oscillation. | 180 | Not specified. | Requires directionally modulated input asymmetries. | Phase precession > 180 degrees. Rate and phase codes can be independent (Huxter et al., 2003, Schlesinger et al., 2015). |
| *Lengyel et al. 2003* | Pyramidal cell | Interference between oscillations with differing frequencies, dendritically generated speed-  dependent oscillator. | Excitatory drive + reference oscillation. | 360 | Speed modulation of dendritic membrane potential oscillation frequency. | Requires difference between dendritic and somatic oscillations to be 180 degrees on entry into the place field. | Place fields are maintained after abolishing theta (Mizumori et al., 1989, Brandon et al., 2014). Rate and phase codes can be independent (Huxter et al., 2003, Schlesinger et al., 2015). |
| *Leung et al. 2011* | Pyramidal cell | Excitation-  dependence of spike latency, asymmetric input, membrane resonance. | Asymmetric excitatory drive + reference oscillation(s). | < 240 | Timescale of excitatory asymmetric input. | Requires directionally modulated input asymmetries. | Requires asymmetric input. Variable firing rates require spike threshold modification. Phase reversal at end of firing field. |
| *Chance et al. 2012* | Pyramidal cell | Interference between spatially offset, amplitude-  modulated oscillations. | Two amplitude modulated oscillations. | 180,  (or 360 when inputs already precess) | Timescale of two Gaussian input amplitudes. | Requires directionally modulated spatial offsets for input oscillations. | Phase precession is maintained following inactivation of CA3 (Middleton and McHugh, 2016). |
| *Jaramillo et al. 2014* | Upstream excitatory cells | Inheritance. | Phase precessing excitatory drive + reference oscillation. | 180-360 | Inherited from inputs. | Requires directionally modulated phase precessing inputs. | CA1 phase precession requires EC (Schlesinger et al., 2015), but not CA3 input (Middleton and McHugh, 2016), while neurons in layer 3 of MEC do not phase precess (Hafting et al. 2008). |
| *Chadwick et al. (introduced here).* | Interneuron | Pacemaker- entrained,  intrinsically generated interneuron activity. | Theta pacemaker + excitatory drive. | 360 (or integer multiples) | Speed-  modulation of pacemaker amplitude and excitatory drive  (both time-  independent). | Omni-  directional without directionally modulated inputs. | Requires that place field maps are correctly organized, otherwise phase precession occurs without network theta sequences (see Figures 6, 7 and Figure 7-Figure Supplements 1 and 3). |
